# Type 1 Diabetes and Cataracts: Investigating Mediating Effects of Serum Metabolites Using Bidirectional Mendelian Randomization

**DOI:** 10.3390/metabo14110644

**Published:** 2024-11-20

**Authors:** Yumeng Shi, Jingxi Qin, Yankai Li, Jin Yang, Yi Lu

**Affiliations:** 1 Shanghai Key Laboratory of Visual Impairment and Restoration, Eye Institute, Eye and Ear, Nose, and Throat Hospital of Fudan University, 83 Fenyang Road, Shanghai 200031, China; ymshi20@fudan.edu.cn; 2 Zhongshan School of Medicine, Sun Yat-sen University, Guangzhou 510080, China; qinjx9@mail2.sysu.edu.cn (J.Q.); liyk63@mail2.sysu.edu.cn (Y.L.)

**Keywords:** type 1 diabetes, cataracts, mendelian randomization, serum metabolites, genetic analysis

## Abstract

Purpose: To investigate the causal relationship between type 1 diabetes (T1D) and cataracts and to explore the mediating role of serum metabolites. Methods: This study employed bidirectional Mendelian randomization (MR) using genetic variants as instrumental variables to infer causality in both directions: from T1D to cataracts and cataracts to T1D. Genetic data for T1D, its complications, and cataracts were sourced from independent genome-wide association study (GWAS) datasets. A two-step multivariable MR combined with mediation analysis was conducted to evaluate the indirect effects of serum metabolites in the causal pathway from T1D to cataracts. Results: The MR analysis demonstrated a significant causal association between T1D and an increased risk of cataracts (OR = 1.01–1.05; *p* < 0.05). Further analysis showed that patients with T1D complications such as coma, ketoacidosis, nephropathy, and retinopathy exhibited a significantly higher risk of developing cataracts compared to those without complications. Sensitivity analyses upheld the robustness of these findings, with no evidence of heterogeneity or pleiotropy. Additionally, 102 serum metabolites were found to exhibit statistically significant mediation effects on cataract risk, with four (13-HODE + 9-HODE, 2-naphthol sulfate, docosadienoate (22:2n6), and X-12906) showing significant mediation effects. Specifically, 13-HODE + 9-HODE had a protective effect, while the other three metabolites were linked to an increased cataract risk. Conclusions: This study provides strong evidence of a causal link between T1D and cataracts, highlighting the mediating role of specific serum metabolites. These findings underscore the importance of early detection and management of cataracts in patients with T1D and suggest potential therapeutic targets for mitigating cataract risk. Further research should focus on replicating these findings in diverse populations and exploring the underlying metabolic pathways in greater detail.

## 1. Introduction

Type 1 diabetes (T1D) is a chronic autoimmune condition involving the destruction of insulin-producing beta cells in the pancreas, leading to a lifelong dependency on exogenous insulin therapy. T1D not only significantly impacts the daily lives of patients but also increases their susceptibility to a range of complications, including cardiovascular diseases, nephropathy, neuropathy, and retinopathy [1,2]. Among the less frequently studied complications is cataract development, a leading cause of visual impairment worldwide [3].

Cataracts, defined as the clouding of the eye’s lens, can significantly affect the quality of life, particularly among the aging population [4,5]. While aging is the predominant risk factor, other factors such as diabetes, smoking, prolonged exposure to ultraviolet light, and certain medications are also known to contribute to cataract formation [4,5]. In diabetic patients, chronic hyperglycemia is thought to accelerate cataract development through various metabolic pathways, including oxidative stress and the polyol pathway [6,7,8,9]. Cataracts are typically linked to type 2 diabetes due to its higher prevalence in individuals over 60 years old [10,11]. Nevertheless, clinicians often encounter cataract cases in younger patients with T1D, with some instances occurring even before the clinical onset of T1D [12,13,14].

The potential causal relationship between T1D and cataracts remains an area of active research. Traditional observational studies targeting patients with T1D often face challenges in establishing causality due to confounding factors and reverse causation [15,16,17]. Mendelian randomization (MR) provides a powerful alternative by using genetic variants as instrumental variables (IVs) to infer causality between an exposure (T1D) and an outcome (cataracts). This method leverages the random assortment of genes at conception, which is independent of confounding factors, thus mimicking the conditions of a randomized controlled trial [18,19,20].

In this study, we applied MR to investigate the causal relationship between T1D and cataracts. Genetic variants strongly associated with T1D were selected from genome-wide association studies (GWAS) to assess their potential indirect effects on cataract development through serum metabolites. This approach aims to provide robust evidence for the causal pathways linking T1D to cataracts, overcoming limitations inherent in conventional observational studies.

To prevent sample overlap, genetic data for T1D, its complications, and cataracts were sourced from independent GWAS datasets, with the methodology adhering to STROBE-MR guidelines to ensure methodological rigor [21,22]. By employing a two-step multivariable MR and mediation analysis, we aimed to elucidate the role of specific serum metabolites as mediators in the causal pathway from T1D to cataracts [23,24]. This approach not only explores the direct causal relationship but also investigates potential intermediate mechanisms, offering a deeper understanding of the underlying biology. Thus, this study aims to fill a gap in the current literature by providing evidence of a causal relationship between T1D and cataracts while highlighting the potential mediating role of serum metabolites.

## 2. Methods

### 2.1. Study Design

**Figure 1:**
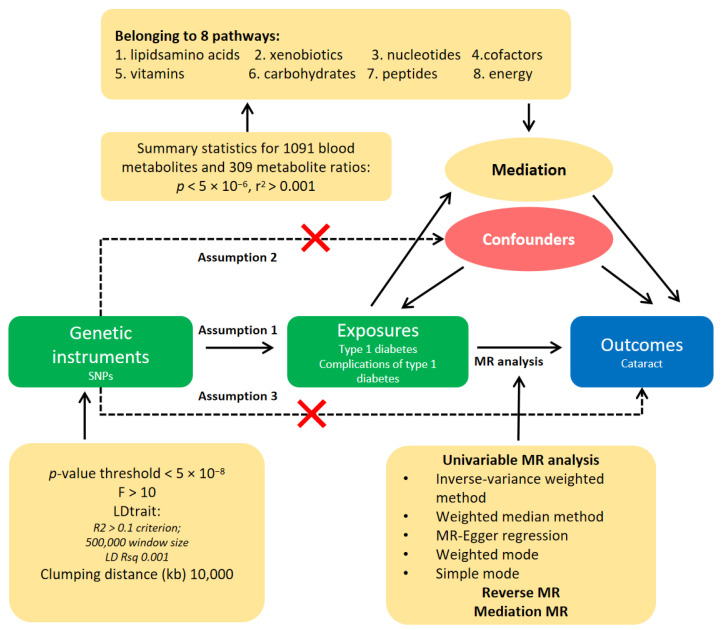
Overview of the study workflow. This diagram illustrates the analytical framework used, starting with the identification of genetic instruments (SNPs) that influence the exposure (type 1 diabetes). Assumptions for Mendelian randomization (MR) are marked as follows: Assumption 1 (genetic variants are associated with the exposure); Assumption 2 (genetic variants are independent of confounders); and Assumption 3 (genetic variants influence the outcome only through the exposure). The exposure to type 1 diabetes, along with its complications, is linked to the outcome of cataract development, mediated by various pathways, including lipid, amino acids, and vitamins. Summary statistics for 1091 blood metabolites across eight metabolic pathways suggest the mediating role of these metabolites. The confounders are indicated as potential biases in the causal interpretation. The MR analysis methods used include univariable approaches such as the inverse-variance weighted method, the weighted median method, MR-Egger regression, and mediation MR, illustrating how each contributes to understanding the causal inference and mediation effects. The figure also denotes the statistical criteria for SNP selection, enhancing clarity on genetic instrument robustness.

Figure 1 illustrates the study design. Genetic variation, allocated randomly before conception, is presumed to remain unaffected by confounding factors other than ancestry, which can be precisely measured and accounted for using genetic data. The Mendelian randomization (MR) analysis was conducted under three fundamental conditions that a genetic variant must satisfy to be an instrumental variable (IV): (1) the selected IVs must be strongly associated with T1D; (2) the selected IVs should not be associated with confounding pathways linked to T1D; and (3) the selected IVs should not affect cataracts directly, only possibly indirectly via T1D. Genetic data for T1D, its complications, and cataracts were obtained from separate, independent genome-wide association study (GWAS) datasets to avoid sample overlap. The analysis adhered to the STROBE-MR (Strengthening the Reporting of Observational Studies in Epidemiology using Mendelian Randomization) guidelines, and the STROBE-MR checklist was completed for this observational study.

### 2.2. Data Sources and Selection of Instrumental Variables

The characteristics of the GWAS data sources are detailed in  Supplementary Material 2 Table S1. We extracted summary-level data on the associations of SNPs with type 1 diabetes (T1D) from three GWAS datasets. GCST010681 is a meta-analysis comprising 9266 T1D cases and 15,574 controls from 12 European cohorts [25]. Dataset GCST90014023 is derived from a large GWAS summary dataset, which includes 520,580 participants (18,942 cases and 501,638 controls) [26], and it was also used for exposure data to identify potential mediating effects of the plasma metabolites. GCST90018925 is derived from a large GWAS summary dataset, which includes 590,946 participants (7666 cases and 583,280 controls) [27]. Data on T1D with complications were obtained from a Finnish database [28]. The cataract data were acquired from the FinnGen consortium (R9), one of the largest medical projects aimed at leveraging genomic information to enhance human health. Notably, when the exposure data on T1D with complications are obtained from the Finnish database, the cataract data are sourced from the UK Biobank (UKB), which includes 456,348 participants (8734 cases and 447,614 controls), to prevent sample overlap. The cataract summary data included 59,522 cases and 312,864 controls. This study defines senile cataract by H25 of the International Classification of Disease-10.

The genome-wide association summary datasets encompassing 1091 blood metabolites and 309 metabolite ratios were derived from the study conducted by Chen et al., representing the most extensive analysis of human metabolites to date [29]. The comprehensive summary statistics are accessible via the Metabolomics GWAS Server. The study analyzed data from 8299 adult individuals enrolled in the Canadian Longitudinal Study on Aging (CLSA) cohort and examined approximately 15.4 million single nucleotide polymorphisms (SNPs). Out of the 1091 plasma metabolites assessed, 850 were identified with known identities across eight superpathways: lipids, amino acids, xenobiotics, nucleotides, cofactors and vitamins, carbohydrates, peptides, and energy. The remaining 241 metabolites were categorized as unknown or partially characterized molecules.

First, SNPs from the GWAS datasets that were significantly (*p* < 5 × 10^−8^) associated with exposures were selected as IVs. Second, we removed SNPs in linkage disequilibrium (R^2^ < 0.001, clumping distance = 10,000 kb) or those that were palindromic with a minor allele frequency above 0.42. Third, we eliminated SNPs with proxy SNPs or those not included in the outcome GWAS. R^2^, the proportion of exposure explained by IVs can be calculated using the formula: R^2^ = 2 × β^2^ × EAF × (1 − EAF), where β is the estimated effect size of the SNPs and EAF is the effect allele frequency. The F-statistic reflects the “strength” of an IV or a set of IVs, defined as the ratio of the mean square of the model to the mean square of the error. F can be calculated by the formula: F = R^2^/(1 − R^2^) × (N − k − 1)/k, where N is the sample size and k is the number of included SNPs. When the F-statistic < 10, we consider the genetic variation used as a “weak IV” (the Staiger–Stock rule), which may produce a certain bias in the results, so SNPs with F-statistic < 10 will be excluded. We extracted IVs of complications of T1D using the same method. Detailed information on those IVs is shown in Supplementary Material 1 Excel 1. Since only a few SNPs were identified for part of the mediators from plasma metabolites when they were used as the exposure, a higher cutoff (*p* < 5 × 10^−6^) was chosen (Supplementary Material 3 Excel 2).

### 2.3. Statistical Analysis

The present study was conducted using R software (version 4.3.2). We utilized several R packages and their associated functions, including base (version 4.2.1), TwoSampleMR (version 0.6.1), MRInstruments (version 0.3.2), MRPRESSO (version 1.0), MendelianRandomization (version 0.6.0), data.table (version 1.15.0), ggplot2 (version 3.5.1), ieugwasr (version 1.0.0), circlize (version 0.4.15), forestploter (version 1.1.1), and LDlinkR (version 1.3.0).

For two-sample MR analysis, we investigated the causal effects of exposure (T1D and its complications, including T1D with coma, T1D with ketoacidosis, T1D with neurological complications, T1D with ophthalmic complications, T1D with renal complications, and T1D with other specified or unspecified multiple complications) on the outcome (senile cataract) using the inverse variance weighted-multiplicative random effects (IVW-MRE) method. The IVW approach is most effective in terms of statistical power when all IVs are valid and there is no horizontal pleiotropy [30]. The effect size is indicated by the odds ratio (OR) along with its 95% confidence interval (CI). We also used the weighted median, simple mode, weighted mode, maximum likelihood, MR Egger, and MR pleiotropy residual sum for additional analysis. Sensitivity analyses were performed to verify and adjust the validity and stability of the results, which included a heterogeneity test (Cochrane’s Q test), pleiotropy test (MR Egger intercept test), and leave-one-out test. Once heterogeneity was identified (*p* < 0.05), the IVW-MRE method should be used for assessing the causal effect. We also performed reverse MR to exclude reverse causal effects.

To comprehensively address confounding, LDtrait was utilized to perform a confounding analysis, focusing on the association between SNPs and identified confounders based on substantial links (R^2^ > 0.1) within a 500,000 base-pair window. This allowed us to identify and adjust for confounders related to both genetic and environmental factors rigorously. By compiling and examining the relevant data regarding SNPs, GWASes, and illnesses, we applied multivariable regression techniques to adjust for these confounders in our statistical models, thereby enhancing the credibility of our findings by reducing potential biases. This process aids in exploring the mediation and possible mechanisms of causation, providing a more robust understanding of the complex interactions at play.

We used summary data from a GWAS of plasma metabolites in 8299 individuals of European ancestry [29] to perform two-step MR to identify potential mediating effects of the plasma metabolites. A total of 1091 blood metabolites and 309 metabolite ratios were included in the analysis. The two-step MR approach was utilized to determine the indirect effect of each mediator. First, we calculated the causative effect of the disease on a potential mediator using IVs for T1D. Second, we determined the mediators’ causal influence on senile cataract using IVs as the mediator. A plasma metabolite was considered a mediator when both MR stages were significant (*p* < 0.05) and overlapped. We calculated the proportion mediated for each mediator separately by dividing the indirect effect by the total effect. The delta approach [31] was utilized to estimate the confidence intervals.

## 3. Results

### 3.1. Selection of IVs

The number of selected instrumental variables (IVs) for the 1091 blood metabolites and 309 metabolite ratios ranged from 4 to 48, with a median of 12 after quality control steps addressing linkage disequilibrium (LD) effects and palindromic SNPs. The median F-statistic was 44.7 (range: 29.9 to 1298.0) for T1D and 22.6 (range: 20.8 to 7180.7) for plasma metabolites. An F-statistic greater than 10 is considered sufficiently informative for MR analyses, suggesting that the likelihood of weak instrumental variable bias is minimal. Detailed information for each SNP, including its R^2^ and F-statistic values, is available in Supplementary Material 1 Excel 1.

### 3.2. MR Analysis

**Figure 2:**
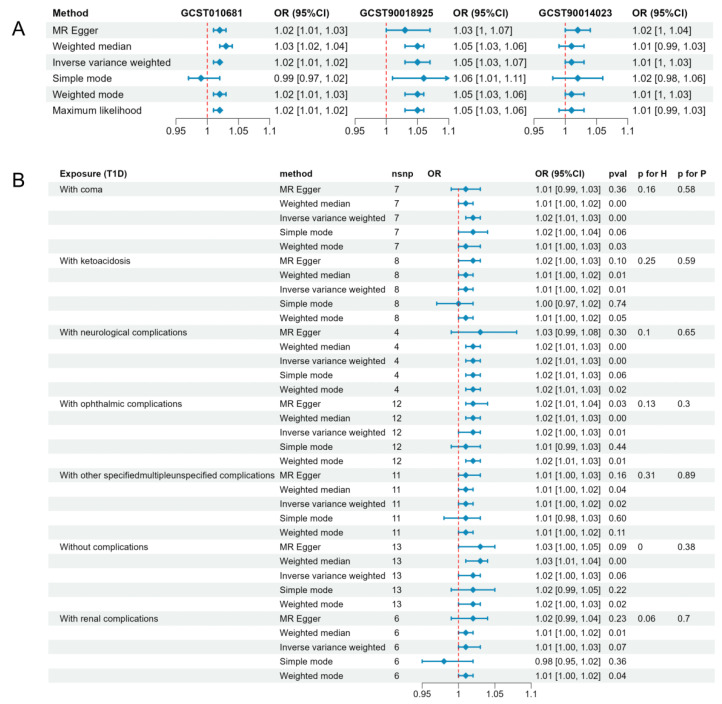
Results of MR analyses conducted to estimate potential associations between T1D and risk of cataract. (**A**) Forest plot depicting the associations between genetically predicted T1D (GCST90014023, GCST010681, GCST90018925) and cataracts across various Mendelian randomization methods, including MR-Egger, weighted median, inverse variance weighted, simple mode, weighted mode, and maximum likelihood. (**B**) Forest plot showing the associations between genetically predicted T1D subgroups, including T1D with coma, ketoacidosis, neurological complications, ophthalmic complications, other specified or multiple unspecified complications, renal complications, and cataracts. Methods used include MR-Egger, weighted median, inverse variance weighted, simple mode, and weighted mode. OR, odds ratio; CI, confidence interval; MR, Mendelian randomization; T1D, type 1 diabetes; H, heterogeneity; P, pleiotropy.

IVW analysis indicated that T1D (IVW: GCST90014023: OR = 1.015; 95% CI = 1.003–1.026; *p* = 0.0124) was causally associated with a significantly increased risk of cataracts in European populations. These findings were consistent with the results from the maximum likelihood method, which showed similar causal estimates in both direction and magnitude (Figure 2A). In the inverse analysis, where senile cataract was used as the exposure and T1D as the outcome, no genetic predisposition to T1D was found to be associated with cataracts (Supplementary Figure S1). Additionally, the results from the MR-Egger, weighted median, weighted mode, and simple mode methods were attenuated (GCST90014023: MR-Egger: OR 1.016; 95% CI 0.996–1.037; *p* = 0.130; Weighted median: OR 1.007; 95% CI 0.990–1.025; *p* = 0.426; Weighted mode: OR 1.010; 95% CI 0.992–1.028; *p* = 0.283; Simple mode: OR 1.017; 95% CI 0.976–1.060; *p* = 0.429) (Table 1).

**Figure 3:**
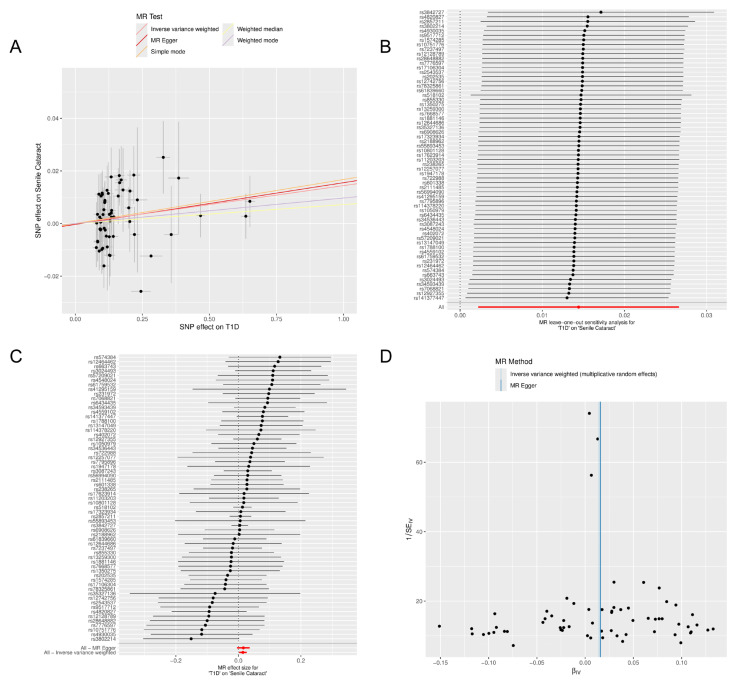
Visualization of MR analyses for the effect of T1D on cataract. (**A**) Scatter plot: Shows the relationship between SNP effects on T1D and cataract. The different slopes of the lines represent the estimated MR effects from various methods, including IVW, MR Egger, simple mode, weighted median, and weighted mode. Each point represents a SNP, with the horizontal axis indicating the SNP’s effect on T1D and the vertical axis indicating its effect on cataract. This plot underscores the consistency of causal estimates across multiple MR methods, highlighting robustness in the face of methodological variations. (**B**) Forest plot (leave-one-out sensitivity analysis): Illustrates the IVW analysis for the impact of T1D on cataract, with each line representing the result when a specific SNP is omitted. The red line at the bottom shows the overall IVW result, including all SNPs. This plot is crucial for assessing the influence of individual SNPs on the overall analysis, ensuring that no single SNP disproportionately affects the outcome. (**C**) Forest plot (effect sizes): Displays MR effect sizes using the MR Egger and IVW methods, showing individual and combined effects of SNPs on the relationship between T1D and cataract. This panel provides insights into the heterogeneity and potential pleiotropy of the instruments used, evidenced by the dispersion and alignment of the estimates. (**D**) Funnel plot: Visualizes the symmetry in the distribution of the SNPs used in the MR analysis. A symmetrical distribution suggests low heterogeneity and pleiotropy among the instruments, reinforcing the reliability of the MR findings; MR, Mendelian randomization; T1D, type 1 diabetes; SNP, single-nucleotide polymorphism; IVW, inverse variance weighted.

No evidence of heterogeneity or pleiotropy was detected in the associations between T1D (GCST90014023) and cataracts (*p* for heterogeneity = 0.783; *p* for pleiotropy = 0.861). Similarly, for GCST010681, no heterogeneity or pleiotropy was observed (*p* for heterogeneity = 0.33; *p* for pleiotropy = 0.07). In contrast, for GCST90018925, heterogeneity was present (*p* for heterogeneity = 0.002), but no pleiotropy was detected (*p* for pleiotropy = 0.266). The robustness of the results was confirmed through leave-one-out sensitivity analysis (Table 2). Detailed data analysis and visual representations, including scatter plots for pleiotropy analysis, forest plots from the leave-one-out method, and funnel plots, are available in Figure 3.

To further explore the relationship between different subgroups of T1D and cataracts, we utilized data from the Finnish database, which offers comprehensive insights into T1D complications. The subgroup analyses indicated significant causal associations between T1D with complications and cataracts. Specifically, the odds ratios (ORs) for cataracts were 1.02 (95% CI 1.01–1.03; *p* = 3.58 × 10^−3^) for T1D with coma, 1.01 (95% CI 1.00–1.02; *p* = 1.41 × 10^−2^) for T1D with ketoacidosis, 1.02 (95% CI 1.01–1.03; *p* = 6.34 × 10^−4^) for T1D with neurological complications, and 1.02 (95% CI 1.00–1.03; *p* = 5.85 × 10^−3^) for T1D with ophthalmic complications (Figure 2B; Table 1). With the exception of the analysis for T1D without complications, where heterogeneity was observed, all other subgroup analyses showed no significant heterogeneity or pleiotropy (Figure 2B; Table 2).

### 3.3. Sensitivity Analysis

Sensitivity analyses were performed to evaluate the robustness of the IVW results. The potential impact of pleiotropy from exposures was considered minimal as no evidence of directional pleiotropy was detected in the MR-Egger regression analysis (*p* > 0.05) (Supplementary Material 1 Excel 1). Cochrane’s Q test also indicated no significant heterogeneity in the MR analysis results between T1D and senile cataract (*p* > 0.05) (Supplementary Material 1 Excel 1).

### 3.4. Confounding Analysis

**Figure 4:**
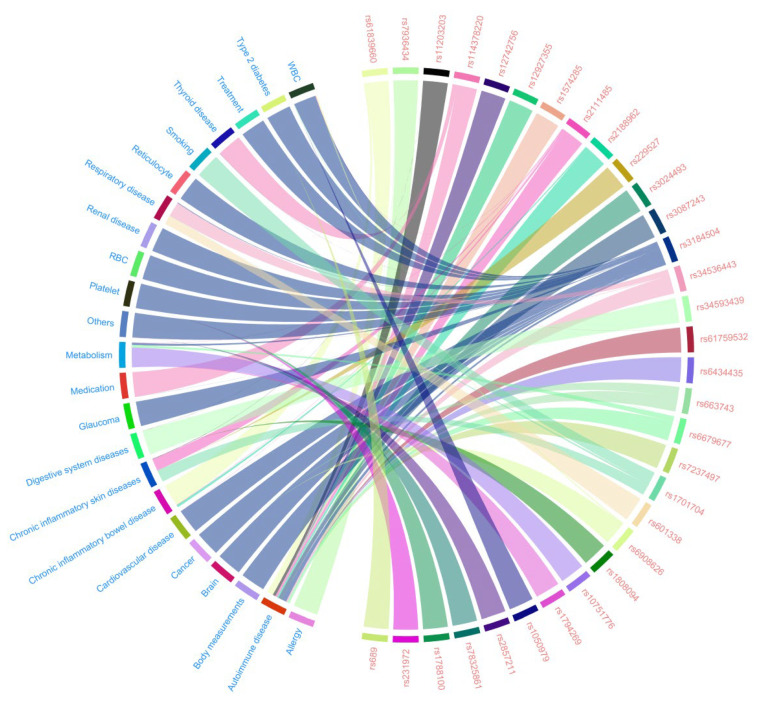
Associations between instrumental variables and potential confounding factors in the confounding analysis. IVs are shown in red, and confounding factors are shown in blue. IVs, instrumental variables; RBC, red blood cell; WBC, white blood cell.

Following a comprehensive review and analysis of relevant data on overweight-associated SNPs, GWASes, and diseases using LDtrait, several potential confounding factors were identified. These included white blood cell (WBC) count, red blood cell (RBC) count, type 2 diabetes, various treatments, thyroid disease, smoking status, reticulocyte count, respiratory diseases, renal diseases, platelet count, metabolic factors, medications, glaucoma, digestive system diseases, chronic inflammatory skin disease, chronic inflammatory bowel disease, cancer, brain metrics, body measurements, autoimmune diseases, and allergies, among others (Figure 4).

### 3.5. Mediation Analyses of Potential Blood Metabolites

**Figure 5:**
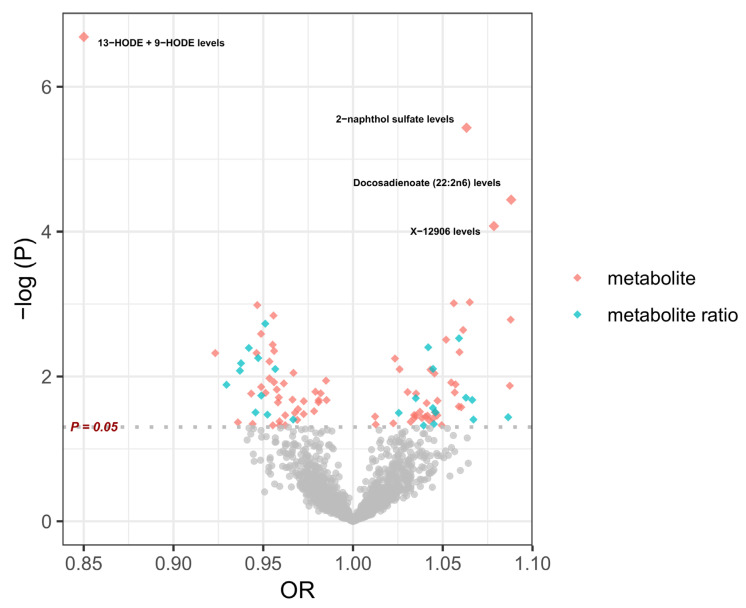
Serum metabolites and metabolite ratios most significantly associated with cataract risk identified by MR analyses. This volcano plot displays the odds ratio (OR) on the *x*-axis and the negative logarithm of the *p*-value (−log(P)) on the *y*-axis, highlighting significant metabolites (red diamonds) and metabolite ratios (blue diamonds). Metabolites positioned to the right of the plot with higher −log(P) values indicate a stronger association with an increased risk of cataracts, while those on the left suggest a protective effect. The horizontal dashed line represents the significance threshold at *p* = 0.05. HODE, hydroxyoctadecadienoic acid; MR, Mendelian randomization; OR, odds ratio.

**Figure 6:**
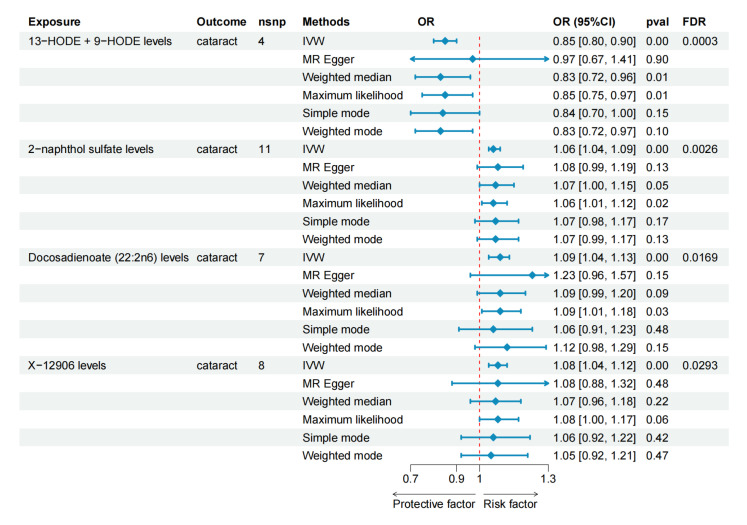
Analysis of causal effects of serum metabolites on cataract using MR analyses. The plot illustrates the associations between four serum metabolites—13-HODE + 9-HODE levels, 2-naphthol sulfate levels, docosadienoate (22:2n6) levels, and X-12906 levels—and cataract risk, as determined by various Mendelian randomization methods, including the inverse variance weighted, MR Egger, weighted median, maximum likelihood, simple mode, and weighted mode. Metabolites positioned to the left of the red dashed line indicate a protective effect, while those to the right suggest an increased risk of cataracts. OR, odds ratio; CI, confidence interval; FDR, false discovery rate; HODE, hydroxyoctadecadienoic acid; MR, Mendelian randomization; IVW, inverse variance weighted.

In the two-step Mendelian randomization (TSMR) analysis, 102 serum metabolites were identified as having a causal relationship with cataracts (*p* < 0.05). Of these metabolites, 50 demonstrated a negative correlation, while 52 showed a positive correlation with age-related cataracts. The four most significant metabolites were 13-HODE + 9-HODE, 2-naphthol sulfate, docosadienoate (22:2n6), and X-12906 (Figure 5; Supplementary Material 3 Excel 2). Their Mendelian randomization results concerning cataract outcomes are shown in Figure 6. Levels of 13-HODE + 9-HODE were found to have a protective effect against cataracts, while levels of 2-naphthol sulfate, docosadienoate (22:2n6), and X-12906 were associated with an increased risk of cataract development (Figure 6).

Among the 102 metabolites, four were found to be causally related to T1D (*p* < 0.05): N-lactoyl valine, trans-urocanate, the alanine-to-pyruvate ratio, and the adenosine 5′-diphosphate (ADP)-to-ethylenediaminetetraacetic acid (EDTA) ratio. Specifically, N-lactoyl valine, trans-urocanate, and the ADP-to-EDTA ratio were negatively correlated with cataracts, whereas the alanine to pyruvate ratio was positively correlated (Table 3). T1D was negatively associated with N-lactoyl valine and trans-urocanate, suggesting a potential reduction in their protective effects against cataracts. Conversely, T1D was positively associated with the alanine to pyruvate ratio, potentially increasing the risk of cataract development. Additionally, T1D exhibited a positive correlation with the ADP to EDTA ratio, which contrasts with its relationship with age-related cataracts (Supplementary Material 3 Excel 2).

## 4. Discussion

In this study, we investigated the causal relationship between type 1 diabetes (T1D) and senile cataracts, specifically focusing on the directionality from T1D to cataract development, and explored the mediating role of serum metabolites. Our results demonstrate a significant causal association between T1D and an increased risk of cataracts. Notably, the reverse MR analysis did not indicate a causal effect of cataracts on T1D, suggesting a unidirectional relationship. Subgroup analyses further demonstrated that patients with T1D with complications—such as coma, ketoacidosis, and neurological, ophthalmic, and other multiple unspecified complications—had a higher risk of developing cataracts. By utilizing genetic variants as instrumental variables, we clarified the directional relationship, addressing potential biases from confounding factors and reverse causation typically encountered in traditional observational studies.

These findings are particularly noteworthy because, while most existing research focuses on the link between type 2 diabetes and cataracts, the relationship between type 1 diabetes and cataracts remains underexplored [32]. This gap in the research is especially significant given that patients with type 1 diabetes tend to experience more severe postoperative outcomes following cataract surgery [33]. Since type 1 diabetes is an autoimmune disease, the immune cascade it triggers may contribute to cataract formation through mechanisms beyond the metabolic changes in aqueous humor circulation solely caused by elevated blood glucose levels [34,35]. Clinically, individual patient factors—such as varying levels of blood glucose control, degrees of glycemic fluctuation, methods of glycemic management, and differences in medication—can significantly impact clinical trial outcomes [36,37], making it challenging to isolate the specific effect of T1D on cataract risk. By employing the Mendelian randomization method, we effectively eliminated these confounding factors, allowing for a clearer exploration of the causal impact of T1D on cataract development.

Our study identified several serum metabolites that mediate the relationship between T1D and cataracts, providing valuable insights into the metabolic pathways involved. Notably, 13-HODE, 9-HODE, docosadienoate, and 2-naphthol sulfate emerged as key metabolites in this process. Both 9-HODE and 13-HODE, primarily derived from linoleic acid via lipoxygenase activity or non-enzymatic processes, have been shown to influence macrophage activity [38]. Additionally, lower plasma levels of these metabolites have been observed in patients with amyotrophic lateral sclerosis compared to healthy controls [39]. Despite these findings, the role of 9-HODE and 13-HODE in age-related cataract development remains unclear, although they are linked to oxidative stress and inflammatory responses—key factors in lens opacification and cataract formation [40]. They act as ligands for peroxisome proliferator-activated receptors (PPARs), which play roles in regulating lipid metabolism and inflammation. Additionally, 13-HODE and 9-HODE can exert pro-inflammatory effects by acting through G protein-coupled receptor 132 (GPR132) [40]. The biochemical pathways involving these metabolites suggest their potential protective or damaging roles through the modulation of oxidative stress mechanisms and inflammation within the lens environment. This can lead to protein aggregation or degradation typical in cataractogenesis. Docosadienoate (22:2n6), a polyunsaturated fatty acid, has primarily been explored in metabolic and cancer research contexts [41,42]. While its specific role in cataract formation is not fully established, there is evidence that alterations in polyunsaturated fatty acids are linked to lens opacity and the progression of cataracts [43,44]. It is hypothesized that docosadienoate may affect cataract development through its impact on cell membrane properties or by influencing inflammatory pathways, which are known factors in cataract pathogenesis. Lastly, 2-naphthol sulfate, a metabolic byproduct commonly associated with the breakdown of naphthalene and other polycyclic aromatic hydrocarbons, has been negatively associated with long-term high red meat consumption in population-based metabolomics studies [45], suggesting its potential role in influencing metabolic health. While it is often examined in the context of environmental exposures and detoxification processes, its specific connection to cataract development remains unclear. Further research is needed to establish the precise pathways through which 2-naphthol sulfate might influence cataract risk.

We also found a significant positive correlation between the alanine-to-pyruvate ratio and T1D. This ratio reflects disruptions in amino acid metabolism that are often observed in patients with T1D, with alanine serving as a key substrate in gluconeogenesis, particularly in the liver [46]. Alanine’s conversion to pyruvate plays a crucial role in glucose recycling between muscles and the liver [47], a process that is frequently impaired in T1D, making it essential for maintaining glucose homeostasis in diabetic conditions [48]. Moreover, elevated alanine levels can increase pyruvate production, subsequently fueling the tricarboxylic acid (TCA) cycle. This intensified metabolic activity raises mitochondrial oxidative phosphorylation demands, potentially leading to an increase in reactive oxygen species (ROS) production [40,49]. Mitochondrial ROS, in turn, contribute to oxidative stress, a critical factor in the development of various diabetic complications, including retinopathy and nephropathy. Persistent oxidative stress, driven by excessive ROS, exacerbates mitochondrial dysfunction and disrupts energy production, thereby promoting inflammation and cellular damage in diabetes [40,50,51,52].

In the diabetic context, oxidative stress also plays a key role in damaging mitochondrial DNA (mtDNA) and impairing mitochondrial function, contributing to the progression of complications such as cataract formation and microvascular damage [52,53,54]. These findings highlight the importance of both amino acid metabolism and mitochondrial health in managing diabetes-related oxidative stress. Further investigation into these metabolic pathways could provide greater clarity on their specific contributions to diabetic complications, including their potential role in increasing the risk of cataract formation. Our Mendelian randomization analysis suggests that targeting these metabolic imbalances could serve as a therapeutic strategy to reduce cataract risk in patients with T1D. By focusing on specific metabolic pathways, it may be possible to develop interventions that alleviate oxidative stress and prevent lens opacity, thereby improving patient outcomes.

To flesh out the clinical value of these findings, it is essential to consider practical guidelines for monitoring and managing cataract development in patients with T1D. Regular ophthalmologic assessments should be integrated into the routine care of these patients to detect early signs of cataract formation. Proactive management strategies, including optimizing glycemic control and addressing modifiable risk, could significantly reduce the risk of cataract development. Furthermore, educating patients about the potential ocular complications of diabetes and the importance of periodic eye examinations could enhance patient outcomes and help in the early detection and treatment of cataracts. Clinicians can also utilize the identified serum metabolites as biomarkers to better understand individual patient risks and tailor interventions accordingly. For instance, monitoring levels of specific metabolites like 13-HODE and 9-HODE may provide insights into the inflammatory status and oxidative stress levels in patients, enabling more targeted therapeutic interventions to mitigate these risk factors.

This study has several limitations that should be considered when interpreting the findings. First, the data used in this analysis are primarily from European populations, which may limit the applicability of the results to other racial or ethnic groups. Genetic variations and metabolic pathways can differ significantly across populations, and further validation in more diverse cohorts is needed to enhance the generalizability of our findings. Replication of this analysis in different ethnic and demographic populations would increase the range of generalization of the results and provide a more nuanced understanding of how genetic and environmental factors influence the relationship between T1D and cataracts.

Second, while the Mendelian randomization approach suggests a unidirectional relationship between T1D and cataracts, this inference requires further validation. Cataract development is a complex process influenced by multiple factors, many of which may not be fully accounted for, especially in younger patients with T1D. Longitudinal study designs, which track the effects of glycemic control over time, are crucial for understanding how fluctuations in glucose levels influence cataract progression. Studies examining the specific determinants of glycemic control and their impact on cataract formation could provide more direct answers regarding the appropriate preventive care for this patient population.

Another limitation of our study is the use of Mendelian randomization, which is a gene-based approach. This method does not account for the temporal dynamics between genes, metabolites, and outcomes over time. As such, the effects of longitudinal changes in gene expression and metabolites on cataract development may not be fully captured, limiting the study’s ability to explain the precise temporal mechanisms at play.

Additionally, although several serum metabolites were identified as mediators in the relationship between T1D and cataracts, the potential for biases in metabolite measurements should be acknowledged. These biases could stem from measurement errors or unaccounted interactions between metabolites and environmental factors. If these biases are not adequately controlled, they could distort the accuracy of the estimated mediation effects, leading to biased conclusions.

In conclusion, this bidirectional MR study provides evidence of a causal relationship between T1D and an increased risk of cataracts, with serum metabolites playing significant mediating roles. These findings have important implications for the prevention and management of cataracts in patients with T1D, emphasizing the need for comprehensive metabolic control and regular ophthalmologic assessments. Our study highlights the utility of MR in uncovering causal relationships in epidemiological research, offering valuable insights into the complex interplay between chronic diseases and their complications. Future research should focus on replicating our findings in diverse populations to enhance the generalizability of the results. Additionally, further investigation into the specific metabolic pathways linking T1D and cataracts could provide deeper insights into the underlying biological mechanisms. A practical next step would be to explore other health complications commonly associated with T1D, as well as the role of additional metabolites, to better understand their interrelationship with cataract development. Such research may identify novel therapeutic targets for preventing cataract development in patients with T1D. Longitudinal studies examining the effects of tight glycemic control on the progression of cataracts in patients with T1D would also be valuable.

## Figures and Tables

**Table 1:** Causal effects of T1D and its complications on cataracts using six MR models.

Exposure	MR Methods	nSNP	Beta	OR (95% CI)	*p*-Value
T1D (Finn)	MR Egger	63	0.016	1.016 (0.996–1.037)	0.130
	Weighted median	63	0.007	1.007 (0.990–1.025)	0.426
	Inverse variance weighted	63	0.014	1.015 (1.002–1.027)	0.021
	Simple mode	63	0.017	1.017 (0.976–1.060)	0.429
	Weighted mode	63	0.010	1.010 (0.992–1.028)	0.283
	Maximum likelihood	63	0.014	1.014 (1.002–1.027)	0.022
T1D (GCST010681)	MR Egger	38	0.022	1.023 (1.013–1.033)	7.87 × 10^−5^
	Weighted median	38	0.026	1.026 (1.016–1.037)	6.43 × 10^−7^
	Inverse variance weighted	38	0.015	1.015 (1.008–1.023)	1.30 × 10^−5^
	Simple mode	38	−0.005	0.995 (0.971–1.019)	0.675
	Weighted mode	38	0.022	1.022 (1.013–1.032)	2.17 × 10^−5^
	Maximum likelihood	38	0.015	1.015 (1.009–1.022)	5.37 × 10^−6^
T1D (GCST90018925)	MR Egger	17	0.032	1.032 (1.000–1.066)	0.072
	Weighted median	17	0.046	1.047 (1.030–1.064)	3.05 × 10^−8^
	Inverse variance weighted	17	0.046	1.047 (1.026–1.069)	1.20 × 10^−5^
	Simple mode	17	0.058	1.060 (1.008–1.114)	0.037
	Weighted mode	17	0.045	1.046 (1.029–1.063)	6.27 × 10^−5^
	Maximum likelihood	17	0.047	1.048 (1.033–1.062)	3.92 × 10^−11^
T1D with coma	MR Egger	7	0.010	1.010 (0.990–1.031)	0.358
	Weighted median	7	0.014	1.014 (1.004–1.024)	0.004
	Inverse variance weighted	7	0.015	1.015 (1.005–1.026)	0.004
	Simple mode	7	0.022	1.022 (1.003–1.042)	0.060
	Weighted mode	7	0.015	1.014 (1.005–1.025)	0.028
T1D with ketoacidosis	MR Egger	8	0.015	1.015 (1.000–1.031)	0.104
	Weighted median	8	0.012	1.012 (1.003–1.022)	0.007
	Inverse variance weighted	8	0.012	1.012 (1.002–1.021)	0.014
	Simple mode	8	−0.004	0.996 (0.974–1.019)	0.738
	Weighted mode	8	0.012	1.012 (1.002–1.022)	0.048
T1D with neurological complications	MR Egger	4	0.031	1.032 (0.988–1.078)	0.296
	Weighted median	4	0.021	1.021 (1.012–1.030)	3.65 × 10^−6^
	Inverse variance weighted	4	0.020	1.020 (1.009–1.032)	0.001
	Simple mode	4	0.020	1.021 (1.007–1.034)	0.060
	Weighted mode	4	0.021	1.021 (1.012–1.030)	0.020
T1D with ophthalmic complications	MR Egger	12	0.024	1.024 (1.005–1.043)	0.032
	Weighted median	12	0.016	1.016 (1.005–1.027)	0.004
	Inverse variance weighted	12	0.015	1.015 (1.004–1.026)	0.006
	Simple mode	12	0.007	1.007 (0.989–1.026)	0.441
	Weighted mode	12	0.017	1.017 (1.006–1.028)	0.014
T1D with other specified multiple unspecified complications	MR Egger	11	0.012	1.012 (0.996–1.028)	0.165
	Weighted median	11	0.011	1.011 (1.001–1.022)	0.039
	Inverse variance weighted	11	0.011	1.011 (1.002–1.021)	0.023
	Simple mode	11	0.006	1.006 (0.984–1.029)	0.603
	Weighted mode	11	0.010	1.010 (1.000–1.021)	0.112
T1D without complications	MR Egger	13	0.026	1.026 (0.999–1.054)	0.085
	Weighted median	13	0.026	1.026 (1.012–1.042)	4.57 × 10^−4^
	Inverse variance weighted	13	0.016	1.016 (1.000–1.033)	0.057
	Simple mode	13	0.020	1.021 (0.989–1.053)	0.223
	Weighted mode	13	0.019	1.019 (1.004–1.033)	0.025
T1D with renal complications	MR Egger	6	0.016	1.015 (0.994–1.038)	0.226
	Weighted median	6	0.013	1.013 (1.003–1.024)	0.010
	Inverse variance weighted	6	0.012	1.012 (0.999–1.026)	0.070
	Simple mode	6	−0.017	0.984 (0.952–1.016)	0.364
	Weighted mode	6	0.013	1.013 (1.004–1.024)	0.039

MR, mendelian randomization; OR, odds ratio; CI, confidence interval; *p*-value, comparing the differences between the two ethnic groups, based on 2 test or *t*-test, as appropriate; nSNP, number of single-nucleotide polymorphisms; T1D, type 1 diabetes; Finn, FinnGen research project.

**Table 2:** Sensitivity analyses of heterogeneity and pleiotropy assessment for MR results.

Exposure	Outcome	Heterogeneity		Pleiotropy
		MR-EggerQ (*p*-Value)	IVW Q (*p*-Value)	Egger_Intercept (*p*-Value)
T1D (Finn)	Cataract	53.056 (0.756)	53.087 (0.783)	−0.000 (0.861)
T1D (GCST010681)		36.573 (0.442)	40.217 (0.330)	−0.004 (0.066)
T1D (GCST90018925)		33.757 (0.004)	36.763 (0.002)	0.006 (0.266)
T1D with coma		8.575 (0.127)	9.191 (0.163)	0.005 (0.575)
T1D with ketoacidosis		8.538 (0.201)	8.987 (0.254)	−0.004 (0.595)
T1D with neurological complications		5.467 (0.065)	6.217 (0.102)	−0.011 (0.653)
T1D with ophthalmic complications		14.701 (0.143)	16.454 (0.125)	−0.006 (0.301)
T1D with other specified multiple unspecified complications		11.632 (0.235)	11.656 (0.309)	−0.001 (0.894)
T1D without complications		30.158 (0.001)	32.476 (0.001)	−0.006 (0.378)
T1D with renal complications		9.977 (0.041)	10.407 (0.064)	−0.004 (0.699)

T1D, type 1 diabetes; MR, mendelian randomization; *p*-value, comparing the differences between the two ethnic groups, based on 2 test or *t*-test, as appropriate; IVW, inverse variance weighted; Q, Cochran’s Q statistic; Finn, FinnGen research project.

**Table 3:** Two-step MR mediation analysis of the association between T1D (exposure) and cataracts (outcome).

Exposure	Outcome	MR Method	nSNP	Beta	Se	*p*-Value
T1D	Cataract (Finn)	IVW	63	0.014	0.006	0.012
T1D	N-lactoyl valine levels		77	−0.023	0.009	0.010
T1D	Trans-urocanate levels		74	−0.021	0.009	0.023
T1D	Alanine to pyruvate ratio		71	0.021	0.008	0.006
T1D	Adenosine 5′-diphosphate (ADP) to EDTA ratio		74	0.025	0.013	0.050
N-lactoyl valine levels	Cataract (Finn)		8	−0.028	0.013	0.033
Trans-urocanate levels	Cataract (Finn)		11	−0.033	0.015	0.032
Alanine to pyruvate ratio	Cataract (Finn)		9	0.065	0.032	0.039
Adenosine 5′-diphosphate (ADP) to EDTA ratio	Cataract (Finn)		9	−0.044	0.017	0.008

T1D, type 1 diabetes; MR, mendelian randomization; nSNP, number of single-nucleotide polymorphisms; Se, standard error; IVW, inverse variance weighted (multiplicative random effects); *p*-value, comparing the differences between the two ethnic groups, based on 2 test or *t*-test, as appropriate; Finn, FinnGen research project; EDTA, Ethylenediaminetetraacetic acid.

## Data Availability

Data used in this study are detailed in Supplementary Material 2 Table S1, which describes the publicly available database accessed.

## Supplementary Materials

The following supporting information can be downloaded at https://www.mdpi.com/article/10.3390/metabo14110644/s1: Supplementary Material 1 Excel 1; Supplementary Material 2 Table S1; Supplementary Material 3 Excel 2; Supplementary Figure S1. Results of the reverse MR study between cataract and risk of T1D. (A) Forest plot of eight MR estimators of the effect of cataract on T1D. (B) Scatter plot of genetic correlation between cataract and T1D using eight MR methods. A evaluation the effect of cataract on T1D. (C) Funnel plots of the association between cataract and T1D. The dark blue line represents (A) MR-Egger and the light blue line represents IVW. (D) MR leave-one-out analysis for cataract and T1D. (E) MR effect forest plot using IVW (multiplicative random effects) and MR-Egger for a single SNP. T1D, type 1 diabetes; OR, odds ratio; CI, confidence interval; MR, mendelian randomization; IVW, Inverse variance weighted.
