# Supplementary material for: Type 1 Diabetes and Cataracts: Investigating Mediating Effects of Serum Metabolites Using Bidirectional Mendelian Randomization
Source: Metabolites. 2024 Nov 20;14(11):644. doi: 10.3390/metabo14110644 (PMC11596039; doi:10.3390/metabo14110644)
Supplement: Supplementary file 1 [file metabolites-14-00644-s001.zip › Supplementary Material 2 Table S1.pdf]

Supplementary Table S1: Information on datasets included in this study.

Phenotypes/ID	Data source	Study information/PMID	Cases/controls	Author/Year
Tyep 1 diabetes; GCST90014023	NHGRI-EBI GWAS	European/34012112	18,942/501,638	Chiou J/2021
Type 1 diabetes: ebi-a-GCST010681	GoKinD,WTCCC#	12 European cohorts/32005708	9266/15574	Forgetta V/2020
Type 1 diabetes: ebi-a-GCST90018925	UKB, Finnish database	European/34594039	6447/451248	Sakaue S/2021
Type 1 diabetes without complications finn-b-E4_DM1NOCOMP	Finnish database	European	8558/392423	NA/2023
Type 1 diabetes with renal complications finn-b-E4_DM1REN	Finnish database	European	1579/392423	NA/2023
Type 1 diabetes with ketoacidosis finn-b-E4_DM1KETO	Finnish database	European	2102/392423	NA/2023
Type 1 diabetes with coma finn-b-E4_DM1COMA	Finnish database	European	2050/392423	NA/2023
Type 1 diabetes with neurological complications finn-b-E4_DM1NEU	Finnish database	European	1077/392423	NA/2023
Type 1 diabetes with ophthalmic complications finn-b-E4_DM1OPTH	Finnish database	European	5202/392423	NA/2023
Type 1 diabetes with other specified/multiple/unspecified complications finn-b-E4_DM1NASCOMP	Finnish database	European	6234/392423	NA/2023

Genomic atlas of the plasma metabolome:

The GWAS summary statistics were deposited to GWAS catalog (https://www.ebi.ac.uk/gwas/). Accession numbers for European GWASs: GCST90199621-90201020; accession numbers for non-European GWASs: GCST90201021-90204063. Individual-level data are available from the Canadian Longitudinal Study on Aging (www.clsa-elcv.ca) for researchers who meet the criteria for access to de-identified CLSA data.

HMDB (http://www.hmdb.ca/system/downloads/current/serum_metabolites.zip).

Finnish database disease inclusion criteria：

Type 1 diabetes with renal complications

Hospital Discharge: ICD-10 E10.2+, E10.2+N08.39

Hospital discharge: ICD-9 2503B

Cause of death: ICD-10 E10.2+, E10.2+N08.39

Cause of death: ICD-9 2503B

Type 1 diabetes with ketoacidosis

Hospital Discharge: ICD-10 E10.1

Hospital discharge: ICD-9 2501B

Cause of death: ICD-10 E10.1

Cause of death: ICD-9 2501B

Type 1 diabetes with coma

Hospital Discharge: ICD-10 E10.00, E10.01, E10.09

Hospital discharge: ICD-9 2502B

Cause of death: ICD-10 E10.00, E10.01, E10.09

Cause of death: ICD-9 2502B

Type 1 diabetes with neurological complications

Hospital Discharge: ICD-10 E10.4+, E10.4+G59.0, E10.4+G63.2, E10.4+G73.0, E10.4+G99.0

Hospital discharge: ICD-9 2505B

Cause of death: ICD-10 E10.4+, E10.4+G59.0, E10.4+G63.2, E10.4+G73.0, E10.4+G99.0

Cause of death: ICD-9 2505B

Type 1 diabetes with ophthalmic complications

Hospital Discharge: ICD-10 E10.3+, E10.3+H28.0, E10.3+H36.09

Hospital discharge: ICD-9 2504B

Cause of death: ICD-10 E10.3+, E10.3+H28.0, E10.3+H36.09

Cause of death: ICD-9 2504B

Type 1 diabetes without complications

Hospital Discharge: ICD-10 E10.9

Hospital discharge: ICD-9 2500B

Cause of death: ICD-10 E10.9

Cause of death: ICD-9 2500B

Type 1 diabetes with other specified/multiple/unspecified complications

Hospital Discharge: ICD-10 E10.6, E10.6+M14.2, E10.6+M14.6, E10.7, E10.8

Hospital discharge: ICD-9 2507B|2508B

Cause of death: ICD-10 E10.6, E10.6+M14.2, E10.6+M14.6, E10.7, E10.8

Cause of death: ICD-9 2507B|2508B
